# A General Linear Relaxometry Model of R_1_ Using Imaging Data

**DOI:** 10.1002/mrm.25210

**Published:** 2014-04-03

**Authors:** Martina F Callaghan, Gunther Helms, Antoine Lutti, Siawoosh Mohammadi, Nikolaus Weiskopf

**Affiliations:** 1 Wellcome Trust Centre for Neuroimaging, Institute of Neurology, University College LondonLondon, United Kingdom; 2 MR Research in Neurology and Psychiatry, Department of Cognitive Neurology, University Medical CenterGoettingen, Germany; 3 LREN, Department des Neurosciences Cliniques, CHUV, Universite de LausanneLausanne, Switzerland

**Keywords:** R_1_, T_1_, PD, PD*, MT, R_2_*, T_2_*, longitudinal relaxation, transverse relaxation, magnetization transfer, quantitative, 3T, water content, relaxometry

## Abstract

**Purpose:**

The longitudinal relaxation rate (R_1_) measured in vivo depends on the local microstructural properties of the tissue, such as macromolecular, iron, and water content. Here, we use whole brain multiparametric in vivo data and a general linear relaxometry model to describe the dependence of R_1_ on these components. We explore a) the validity of having a single fixed set of model coefficients for the whole brain and b) the stability of the model coefficients in a large cohort.

**Methods:**

Maps of magnetization transfer (MT) and effective transverse relaxation rate (R_2_*) were used as surrogates for macromolecular and iron content, respectively. Spatial variations in these parameters reflected variations in underlying tissue microstructure. A linear model was applied to the whole brain, including gray/white matter and deep brain structures, to determine the global model coefficients. Synthetic R_1_ values were then calculated using these coefficients and compared with the measured R_1_ maps.

**Results:**

The model's validity was demonstrated by correspondence between the synthetic and measured R_1_ values and by high stability of the model coefficients across a large cohort.

**Conclusion:**

A single set of global coefficients can be used to relate R_1_, MT, and R_2_* across the whole brain. Our population study demonstrates the robustness and stability of the model. Magn Reson Med, 2014. © 2014 The Authors. Magnetic Resonance in Medicine published by Wiley Periodicals, Inc. **Magn Reson Med 73:1309–1314, 2015. © 2014 Wiley Periodicals, Inc.**

## INTRODUCTION

At a given field strength, the rate of longitudinal recovery (R_1_ = 1/T_1_) is determined by the microstructural tissue environment (eg, the local mobility of water molecules), the macromolecular content, and the local concentration of paramagnetic ions such as iron or gadolinium-based contrast agents. The linear dependence of R_1_ on these tissue properties has been modeled and verified with independent laboratory measures from excised tissue 1–7 or by reference to literature values in the case of iron content 8.

Rapid cross-relaxation with macromolecules facilitates energy exchange between excited protons and their local environment, increasing the observed R_1_ recovery rate. When macromolecular protons are selectively saturated using off-resonance radiofrequency (RF) irradiation, the MR water signal is attenuated by magnetization transfer (MT) 9. In MRI of humans, MT contrast is invoked by application of off-resonant RF pulses prior to excitation. In voxels with a higher macromolecular content, the mobile water will experience a greater percentage loss of signal (MT saturation) 10 as a consequence of a given prepulse and the dynamic of the MT 11. Consequently, measures of MT provide information about the macromolecular content of the microstructural environment and provide us with surrogate markers or model estimates for the bound water fraction 12. In a gradient echo acquisition, the observed MR signal created after RF excitation decays over time by the effective transverse relaxation rate (R_2_* = 1/T_2_*). The presence of paramagnetic metals, including iron, leads to local distortion of the static B_0_ field causing a more rapid decay (ie, higher R_2_*) 13.

In this study, we used a quantitative multiparameter mapping (MPM) protocol to acquire whole brain quantitative maps of the longitudinal relaxation rate (R_1_), effective transverse relaxation rate (R_2_*), and percent saturation due to MT. We used a linear model of R_1_ that is motivated by the linear dependence of the measured R_1_ on the relaxation rates of the individual spin pools under the conditions of fast exchange between pools. The measured MT saturation and R_2_* estimates served as surrogate concentrations for macromolecules and iron, respectively, to calculate the model coefficients. The validity of estimating a single set of model coefficients for the whole brain—including gray matter, white matter, and deep gray matter structures—was assessed by calculating the Pearson coefficient between the measured R_1_ values and synthetic R_1_ values generated using the model coefficients. A set of model coefficients was calculated individually for each of 138 volunteers to examine their stability across a population.

## METHODS

### Data Acquisition

A whole brain quantitative MPM protocol 14 was used to acquire 1-mm isotropic data from 138 healthy volunteers (male, n = 49; female, n = 89; age range, 19–75 y [mean age, 46.6 y; standard deviation, 21 y]) on two 3T whole body MR systems (Magnetom TIM Trio, Siemens Healthcare, Erlangen, Germany, 69 volunteers per scanner) each equipped with a standard 32-channel head coil for receive and RF body coil for transmission. The protocol consisted of PD-, T_1_-, and MT-weighted multiecho FLASH acquisitions and additional B_1_ field calibrations as described by Weiskopf et al. 14. The total scanning time of the MPM protocol was ∼25 min. Informed written consent was obtained prior to scanning with approval from the local ethics committee.

Quantitative maps were derived from the MPM protocol using bespoke MATLAB tools (Mathworks, Natick, Massachusetts, USA). In brief, regression of the log signal from the eight PD-weighted echoes was used to calculate a map of R_2_*. The first six echoes of each of the three acquired weightings were then averaged to increase the signal-to-noise ratio 15. The resulting PD-weighted, T1-weighted, and MT-weighted volumes were used to calculate maps of MT and R_1_ as described previously 10,14,16. To maximize the accuracy of the R_1_ maps, inhomogeneity in the flip angle was corrected by mapping the B_1_^+^ transmit field according to the procedure detailed in the study by Lutti et al. 17 and the intrinsically imperfect spoiling characteristics were corrected using the approach described by Preibisch and Deichmann 18. Use of the measured R_1_ maps in in vivo histological studies illustrated the high level of accuracy of the technique 19–21.

The semiquantitative MT map depicts the specific percentage loss of magnetization caused by a Gaussian RF pulse (4 ms duration, 220° nominal flip angle) applied 2 kHz off-resonance prior to nonselective excitation. This differs from the commonly used MT ratio (MTR, percentage reduction in steady state signal) by explicitly accounting for spatially varying T_1_ relaxation times and flip angles 10 and results in higher contrast in the brain than the MT ratio 22. Additional minor corrections for flip angle inhomogeneity in the MT maps were applied as described by Weiskopf et al. 14.

### Linear Relaxometry Model

Under the conditions of fast exchange, the cross-relaxation time between different water components is assumed to be much shorter than the MR relaxation times. In this case, the measured longitudinal relaxation rate is a weighted sum of the relaxation rates of the various contributory components 7:


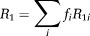
[1]

where *f_i_* is the fraction of spins in pool *i* with relaxation rate *R*_1*i*_. In the absence of any exogenous contrast agents, the measured R_1_ will predominantly depend on the fraction of free water spins, as well as the fraction of bound water spins at macromolecular sites and, on a smaller contribution, from iron sites 8,23. In this case, equation [1] becomes:


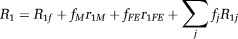
[2]

where *R*_1*f*_ is the relaxation rate of free water; *f_M_* is the fraction of spins bound to macromolecules; *r*_1*M*_ is the relaxivity at macromolecular sites (ie, *R*_1*M*_ − *R*_1*f*_) where *R*_1*M*_ is the relaxation rate at macromolecular sites, *f_FE_* is the fraction of spins at iron sites, and *r*_1*FE*_ is the relaxivity at iron sites; the index *j* sums over all potential unspecified contributions that remain.

### Estimating Model Parameters

A model of R_1_ based purely on imaging data can be constructed from Eq. [2] by replacing the known contributors to R_1_ with surrogate markers. Using the MT and R_2_* maps from the MPM protocol as surrogate markers for the macromolecular and iron concentrations, respectively, the model can be expressed as a function of spatial position, *r*, as:


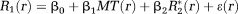
[3]

**Figure 1:**
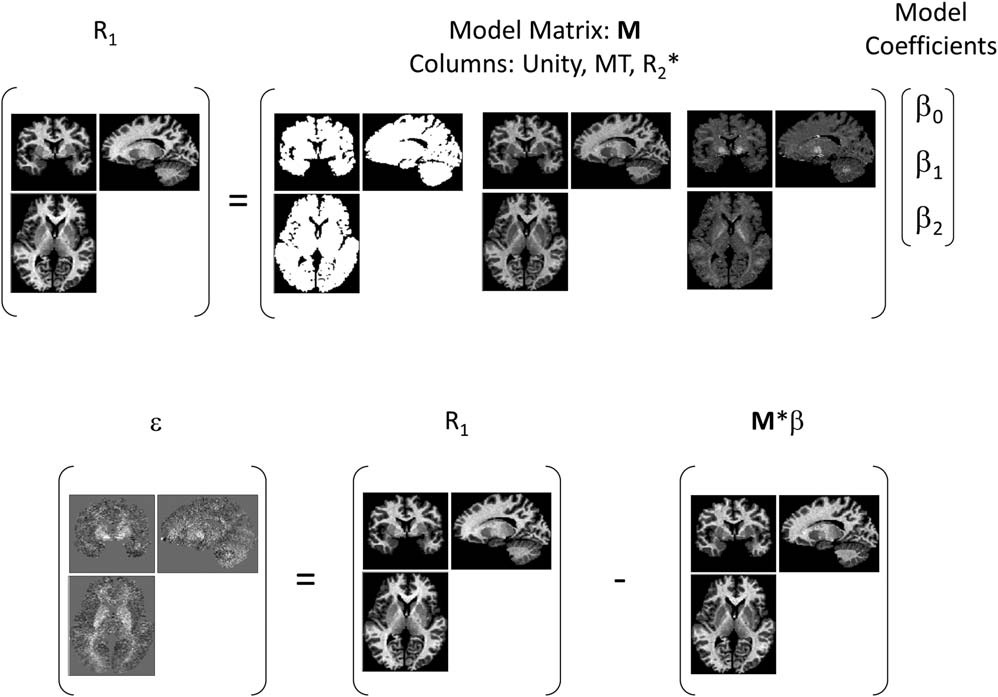
Illustration of the linear relaxometry model that is constructed on a per-subject basis. The three model coefficients are the least squares solution to the matrix equation R_1_ = Mβ.

Here the set of β parameters are global constants and ε(*r*) is the spatially specific residual encompassing the unspecified contributions to R_1_ and noise. For a given subject, a model matrix, **M**, is constructed with three columns: unity, MT, and R_2_* (Fig. 1). In this case, the β parameters are the model coefficients and least squares solution to the matrix equation R_1_ = **M**β, and the residuals are the difference between the measured and synthetic R_1_ values (ie, ε = R_1_ − **M**β).

The parameters of the linear relaxometry model were estimated for each volunteer separately by fitting the relationship across all brain voxels (excluding cerebrospinal fluid, which does not contain appreciable amounts of macromolecules or iron and is not well-characterized by the particular acquisition protocol used in this study). Brain voxels were identified by automated segmentation using SPM8 24. All voxels with a gray or white matter probability >50% and a cerebrospinal fluid probability <50% were pooled to estimate the global, tissue-independent β parameters as well as a spatial map of the residual error on a subject-by-subject basis. The accuracy of the model was assessed by calculating a synthetic R_1_ map from the β parameters (ie, **M**β). The Pearson coefficient between the synthetic and measured R_1_ values was calculated for each subject.

## RESULTS

**Figure 2:**
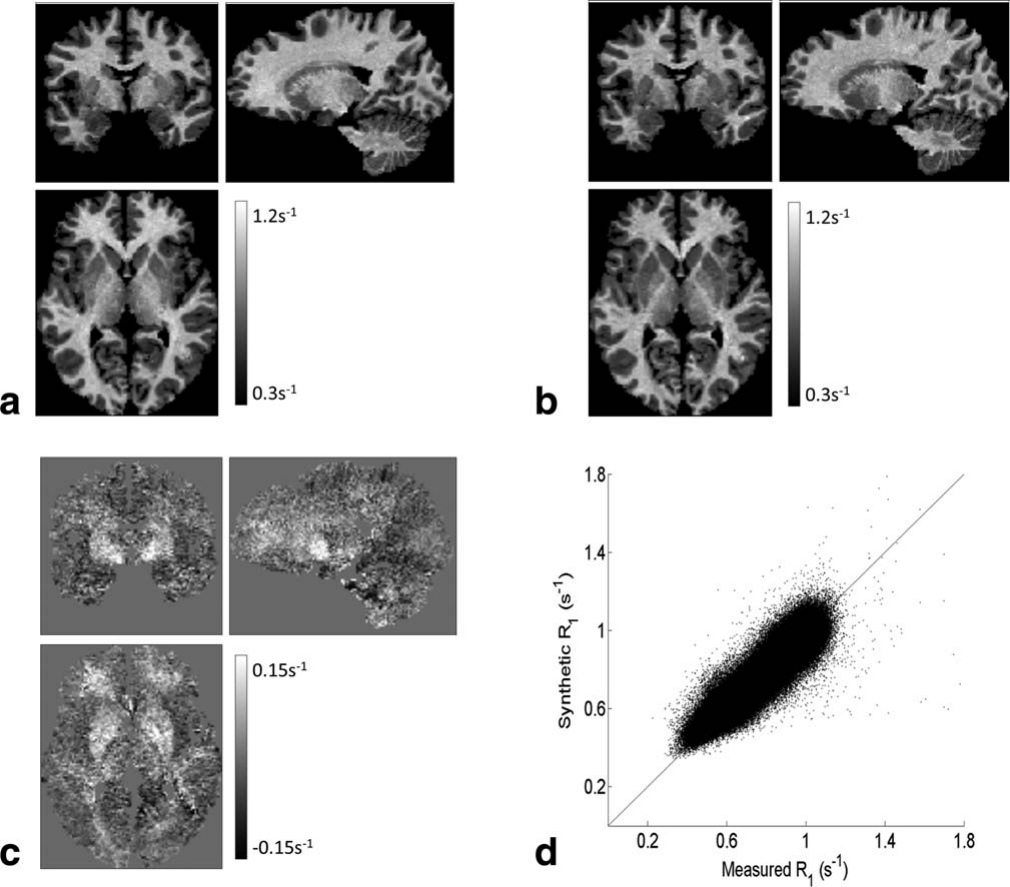
Exemplary single subject data from the cohort using a masking threshold of 30% on the tissue probabilities. The global β coefficients for this subject were: 0.2692 s^−1^, 0.3979 s^−1^/p.u., and 0.0011. The Pearson coefficient of the model in this subject was 0.96. (a) Measured R_1_ map. (b) R_1_ map synthesized using the model coefficients. (c) Spatial map of the model residuals (ie, the difference between the measured and synthesized R_1_ maps). (d) Synthesized R_1_ values plotted against the measured R_1_ values across the whole brain illustrates the high correspondence between the two R_1_ measures.

A single subject example data set is shown in Fig. 2 along with the model estimated R_1_ and the residuals (ie, the difference between the estimated and measured R_1_ values). The linear model fitted well with a mean Pearson coefficient across the entire cohort of 0.93 ± 0.03. The resulting β parameters are summarized for the group in Table1. The coefficient of variation across volunteers was <5.5% for both β_0_ and β_1_ but rises to 37.2% for β_2_.

**Table 1:** Summary Statistics for the Global Parameters of the Linear Model Across the Cohort at a Threshold of 50%

Parameter	Mean	Standard Deviation	Coefficient of Variation
β_0_ (s^−1^)	0.2677	0.0142	5.32%
β_1_ (s^−1^/p.u.)	0.3971	0.0184	4.64%
β_2_	0.0025	0.0009	37.15%

The residuals were generally close to or within the acquisition noise level 14. Within the whole brain, they are centered at −0.13 ± 0.34% across the cohort. Examining the residuals by tissue class revealed a bias of 1.62 ± 0.38% toward lower model R_1_ values in white matter and a bias of 0.97 ± 0.52% toward higher model R_1_ values in gray matter. The residual maps show that the greatest deviation of the model to the measured R_1_ occurred within the basal ganglia, where it was underestimated by the model to varying degrees but in the order of 10%.

The β_0_ parameter corresponded directly to the relaxation rate of free water. The estimated longitudinal relaxation time (T_1_) of free water in brain tissue was 3.736 ± 0.198s.

## DISCUSSION

A single set of β parameters sufficed to model R_1_ across the whole brain, incorporating voxels from both gray and white matter. The model parameters describing the influence of free water and MT on R_1_ were highly stable across this large cohort, suggesting that underlying microstructural differences due to macromolecular content are well captured by the MT map across both gray and white matter. This may be due to the fact that the transfer times are similar in gray and white matter 25. In agreement with the well-established two-site-fast-exchange model 2,7,23,26, the contribution from macromolecular components has been shown to be the dominant factor within the model. In addition, it has been shown that when gray and white matter are considered concurrently water content is a better predictor of R_1_ variation than is iron content 8. The β_0_ parameter appears to give a reliable estimate of the longitudinal relaxation rate of free water (R_1f_) that is in keeping with values reported in the literature [see Table1 in Rooney et al. 23]. The remaining components of the model contain inseparable components: the fraction of the spins at the site and the relaxivity of the site. For example, the β_1_MT term of the model corresponds to the product of the macromolecular bound water fraction and the relaxivity at these macromolecular sites. Either of these components may change depending on the local microstructure. To separate the contributions and validate this model further, one or the other factor must be known. The bound water fraction can be measured histologically using calorimetric approaches, as has been done for white matter 3. Using the histological estimate of water fraction and combining it with our results yields a relaxivity estimate of 3.687 ± 0.198s^−1^ for WM, which is in good agreement with the literature 23.

**Figure 3:**
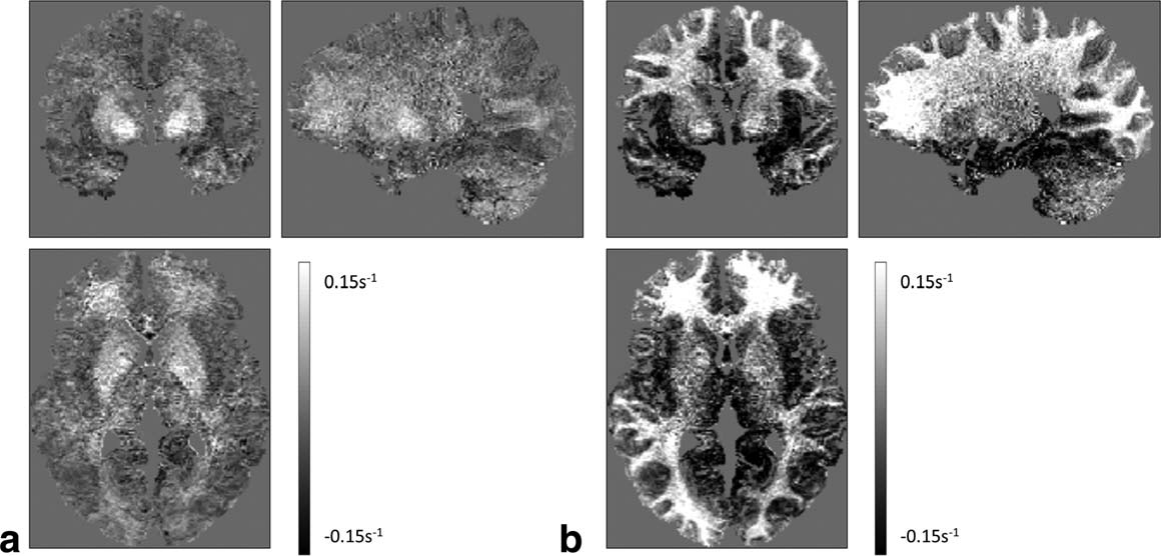
Residuals from the linear model were significantly lower and contained far fewer anatomical structure and bias field effects when MT (a) rather than MTR (b) was used as a surrogate for macromolecules.

Post mortem validation has shown high correspondence between R_2_* and iron content 13 and between MTR and myelin content 12. The fact that the β parameters largely showed good stability across this large cohort (age range, 19–75 y) validates our use of MT and R_2_* as surrogate markers for macromolecular and iron content. When the MTR measure was used in the model, the model coefficients were less stable and the model residuals were increased with anatomical and bias field structure present (Fig. 3). The stability of the model coefficients also suggests that they could be used to generate an estimated R_1_ map directly from MT and R_2_* maps. Applying the model in such a way would facilitate a reduction in the total scan time for the quantitative MPM approach. It would also allow a synthetic quantitative map to still be calculated from suboptimal data (eg, if motion occurred during part of the protocol). The volunteers included in this analysis were all healthy volunteers with no evidence of cognitive impairment. Because we did not study patients, we cannot conclude whether a different set of model parameters may be required in pathological conditions. If they were, then the resulting parameters may become a diagnostic measure.

**Figure 4:**
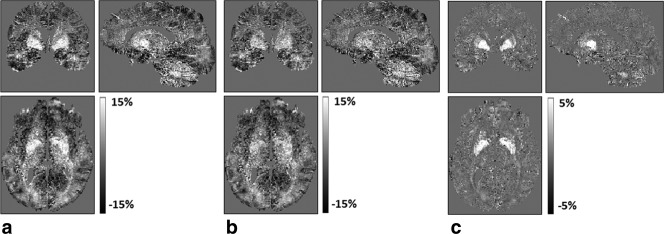
Normalized residuals expressed in percent units from the linear model incorporating free water and macromolecular content (a) were reduced by also including an iron term (b). (c) The difference highlights iron-rich structures, such as the pallidum and dentate nucleus, in which the residuals were particularly reduced.

The model parameter describing the R_1_ dependence on R_2_* (ie, β_2_) had a markedly higher coefficient of variation across volunteers than the free water or macromolecular terms. For the majority of the brain, the model performs well without the inclusion of the iron term, and the Pearson coefficient remains high (mean, 0.92). However, inclusion of the iron term via the R_2_* measure reduces the residuals for high iron structures, such as the basal ganglia and dentate nucleus (Fig. 4). This is to be expected, since it is in these structures that iron will contribute most significantly to R_1_. Nonetheless, the residuals remain highest in these structures, suggesting that the iron effects are not described fully by the model. Additional or higher-order terms (eg, accounting for the different chemical forms in which iron is present in vivo 27), may be required to more fully model the true R_1_ variation in these structures. Additionally, the comparatively smaller contribution from iron sites may be poorly estimated because of the higher noise level in the R_2_* map estimated by our MPM protocol which uses a maximal echo time of 19.70 ms and because the multiecho three-dimensional FLASH volumes used to calculate the MT and R_1_ maps are averaged resulting in an effective echo time of 8.45 ms and therefore residual R_2_* weighting. It has also been suggested that the measured R_2_* will depend on the orientation of white matter fibers with respect to the main field 28–30. These effects are currently not accounted for, though it may be possible to extend the model to include them.

The residuals of the model fit can have a variety of sources, typically falling into two categories: unknown or unspecified model terms, as discussed above, and noise. In some cases, the residuals are high because they capture a spatially coherent form of noise (ie, artifact) present in one or more of the maps used in the model. This is most typically caused by subject motion during the acquisition of one of the constituent three-dimensional FLASH volumes of the MPM protocol. Because the volumes combine in different ways to calculate each of the quantitative maps such motion can lead to inconsistencies across the model matrix components, which may in turn be captured by the residuals. This feature raises the interesting possibility of using the general linear relaxometry model for artifact characterization and correction. Many opportunities arise from the application of this model such as further understanding R_1_ dependencies, potential disease biomarkers, and artifact correction.

## CONCLUSIONS

We have shown that a general linear model of longitudinal relaxation can be applied voxel-wise across the whole brain by using MT and effective transverse relaxation maps as surrogate concentrations for macromolecules and iron, respectively. This model fits well and provides a single set of model parameters per individual that is remarkably stable across the cohort.
